# A Critical Catalogue of the Works Which Have Been Published on Mineral Waters in General, and Particularly Those of France; &c.

**Published:** 1799-05

**Authors:** 


### CRITICAL RETROSPECT OF *MEDICAL AND PHYSICAL LITERATURE.*

### [FOREIGN AND DOMESTIC.]

### NATURAL HISTORY AND CHEMISTRY.

*Catalogue raiſonné des ouvrages, &c.—A critical catalogue of the works which have been publiſhed on mineral waters in general, and particularly thoſe of France;* &c. By Cit. CARRERE, 4to. 400 pp. (10 livres) *Paris.* Rémont.

This valuable *monographical* book is divided into four diſtinct parts; in the firſt, we find a liſt of works on mineral waters in general, containing the titles together with an analyſis of *two hundred* and *fifty-two* publications on that ſubject;—the ſecond relates to the mineral waters of France in particular, and preſents an analytical view of *nine hundred* and *two* works, with an account of *ſix hundred* and *twenty-ſeven* ſprings;—the third gives a ſuccinct liſt of the mineral ſprings of France, not before deſcribed, and which amount to the almoſt incredible number of four hundred and forty-ſeven;—laſtly, the fourth ſection of the book contains tables, ſtating the temperature of the hot ſprings of France, compared with that of the atmoſphere.

The preſent is a new edition of a work, which has formerly been received with great approbation by medical men, as well as general readers; it was originally publiſhed under the patronage of the ci-devant Royal Society of Medicine, in conſequence of a favourable report made of it, by the late celebrated Vic. d'Azyr.

